# Editorial: Advances in the management of tuberculosis meningitis

**DOI:** 10.3389/fimmu.2024.1433345

**Published:** 2024-06-12

**Authors:** Selvakumar Subbian, Vishwanath Venketaraman

**Affiliations:** ^1^ Public Health Research Institute Center at New Jersey Medical School, Rutgers University, Newark, NJ, United States; ^2^ Department of Basic Medical Sciences, College of Osteopathic Medicine of the Pacific, Western University of Health Sciences, Pomona, CA, United States

**Keywords:** tuberculosis meningitis, diagnosis, pathogenesis, mycobacteria, immunity, cerebrospinal fluid, host response

Bacterial meningitis (BM), including tuberculous meningitis (TBM), is a major health concern globally, with a high mortality rate, particularly among children and vulnerable populations (WHO report, 2023; https://www.who.int/news-room/fact-sheets/detail/meningitis). Meningitis affects the brain and spinal cord and can occur as a secondary infection in conditions such as sepsis. Clinical features of BM include persistent fever, confusion, headache, neck stiffness, cerebral infarcts, and mass lesions (1). Similarly, TBM, the most severe form of TB, is described as the manifestation of *Mycobacterium tuberculosis* infection in the meninges, leading to inflammation and disease that causes death in approximately 25% of cases despite antibiotic therapy, and half of survivors are left with neurological disability. Delay in diagnosis and treatment of meningitis results in either rapid death or substantial permanent neurological morbidity (2). However, due to its broad and non-specific clinical spectrum of symptoms, meningitis remains challenging to diagnose and treat (1). Stroke occurs in 45% of patients with TBM in both early and late stages, mainly in the basal ganglia region, and predicts poor outcomes three months after onset (3). In addition, individuals with weakened immune systems, particularly those infected with the human immunodeficiency virus (HIV), are more susceptible to both pulmonary and extrapulmonary secondary bacterial infections in addition to BM and TBM. Since mortality and morbidity in meningitis are contributed to by a dysregulated immune response, adjunctive host-directed therapies are a potential treatment option to modulate this response and improve treatment outcomes. Developing such therapies relies on an improved understanding of the host immune response underlying BM. This Research Topic includes a review and research articles that provide comprehensive information on the latest developments in the understanding of BM and TBM, and management strategies for this deadly disease.

The research article by Ye et al. demonstrates the effects of Ma Xing Shi Gan Decoction (MXSGD) to ameliorate cyclosporine A (CsA)-induced hypo-immune lung injury by regulating microflora metabolism. MXSGD is a traditional remedy for treating lung injury that was developed by the Typhoid and Fever School of Pharmaceutical Biology at the Hunan University of Chinese Medicine, Hunan, China. It has been shown to have antitussive and expectorant effects, along with anti-inflammatory and antiviral properties that regulate host immunity against infections. These study findings indicate that MXSGD was able to preserve lung tissue morphology and structure, reduce the expression of serum inflammatory markers, and protect against CsA-induced lung tissue damage.

Current diagnostic modalities for distinguishing TBM from BM lack sufficient sensitivity and specificity. For example, the conventional microscopic examination for acid-fast bacilli (AFB) on a cerebrospinal fluid (CSF) smear has a sensitivity of approximately 10% (4). Even advanced molecular diagnostic tests such as Xpert TB methods showed only 34%-47% sensitivity (5). Systemic and local inflammation in BM contributes to exacerbated immune cell infiltration at the site of infection, which can be assessed by CSF analysis. However, CSF cytology for the diagnosis of BM is limited by the number and type of inflammatory cells detectable by this simple technique, resulting in a sensitivity of approximately 55%-70% (6). Although flow-cytometric analysis of cellular markers improved the sensitivity of CSF-based diagnosis to approximately 90% success, the availability and compatibility of specific marker panels are critical parameters for the success of this method (6). Xiao et al. applied a high-throughput single-cell RNA-sequencing method to determine the cellular heterogeneity in the CSF of 33 children with different severities of BM, caused by various bacterial pathogens, including *Streptococcus agalactiae*, *Streptococcus pneumoniae*, *Escherichia coli*, *Listeria monocytogenes*, *Enterococcus faecium*, and *Neisseria meningitidis*. The authors identified 18 gene-signature clusters to profile the differential distribution of neutrophils, monocytes, macrophages, dendritic cells, T and B cells, NK cells, pDCs, and plasma cells distributed at different stages of BM with or without desirable treatment outcomes. Further analysis of the data may shed light on the mechanism of pathogenesis and potential therapeutic targets for BM.

In another study, Luo et al. applied a combination of determinations of tuberculosis-specific antigen/phytohemagglutinin (TBAg/PHA) ratio, chlorine level, nucleated cell count, and CSF lymphocyte proportion to differentiate TBM from BM. Using these parameters, the authors achieved approximately 80% sensitivity and 90% specificity in differentiating TBM from BM in two clinical cohorts of patients. However, these findings need to be validated in large cohorts of patients with BM or TBM of differing severities, with and without treatment, before advancing to clinical applications.

The review article by Barnacle et al. describes our current understanding of the human immune response in TBM by discussing Mtb entry into the CNS, microglial infection, and blood-brain and other CNS barrier dysfunction. The review then outlines the innate response, including the early cytokine response, the role of canonical and non-canonical inflammasomes, eicosanoids, and specialized pro-resolving mediators. The review also discusses the adaptive response in TBM, including T cells, microRNAs and B cells, followed by the role of the glutamate-GABA neurotransmitter cycle and the tryptophan pathway. Furthermore, host genetic immune factors, differences between adults and children, paradoxical reactions, and the impact of HIV-1 co-infection including immune reconstitution inflammatory syndrome in addition to promising immunomodulatory therapies, research gaps, ongoing challenges, and future paths are also discussed.

In summary, this Research Topic includes articles that provide valuable information on various aspects of BM management. For example, the biochemical and molecular tools proposed by Xiao et al., and Luo et al. can potentially be used to diagnose TBM and discriminate between BM and TBM, respectively. Similarly, traditional drug formulations, such as MXSGD reported by Ye et al., have the potential to be used as an alternative to allopathic medications to attenuate systemic inflammation and lung injury caused by infectious agents. Thus, the concepts and findings presented in these articles would provide a platform for devising better and improved diagnostic and treatment strategies for BM, as well as for other infectious diseases. However, additional studies on the evaluation of these modalities in relevant preclinical models of BM are warranted before further consideration for clinical applications.

## Author contributions

SS: Writing – original draft, Writing – review & editing. VV: Writing – original draft, Writing – review & editing.
